# Prospective study of 393 adolescent thoracic hyperkyphosis patients treated by the Lyon method

**DOI:** 10.1186/1748-7161-8-S2-O50

**Published:** 2013-09-18

**Authors:** Jean Claude de Mauroy, Didier Fort

**Affiliations:** 1 Clinique du Parc, Lyon, France

## Background

Unlike scoliosis, there is no alteration in respiratory function with hyperkyphosis[1]. The resulting problems are mainly related to aesthetics and pain. The various conservative orthopedic treatments were discussed at the 7th SOSORT consensus session[2]. Is bracing useful to improve aesthetics and prevent back pain? 

## Purpose

A retrospective study was presented at the SOSORT Montreal. [3] The good results observed were complemented by a prospective study performed on our entire database of orthopedic medicine between 1998 and 2007.

## Methods

The Lyon method includes:

- A reduction with plaster cast for a minimum of 1 month to increase the length of the anterior longitudinal ligament (creep).

- An immobilization in a corrected position by a plexidur 5 points brace worn at minimum during the night.

- A specific physiotherapy.

## Results

Conservative treatment was indicated in 393 patients.

- 27% of patients do not accept the proposed treatment or interrupt it spontaneously.

- 23% of patients have a physiological angulation less than 44° at the end of treatment.

- 43% of patients were reviewed two years after removal of the brace. 

The initial kyphosis angle was 60.5°.

The final angulation 2 years after removal of the brace was 41°.

Among the 262 patients who performed the full treatment:

- All patients who had pain before treatment were relieved of that pain after treatment began. 

- 79% were fully corrected with final angulation < 45°. 

- 17% were stabilized with a final angulation between 45° and 55°. 

- 11% retained an angle of > 55° and can be considered as treatment failures. 

In total, 222 patients were reviewed more than 10 years after removal of the brace. The angle remains stable in 21 cases. 

## Conclusions and discussion

The Lyon method is difficult for the patient and one-third of patients did not accept it. 

Unlike scoliosis, which is stabilized by orthopedic treatment, it is possible to restore a physiological kyphosis in the sagittal plane. 
